# Nodal histiocytic sarcoma with prominent eosinophilic infiltration: expression of eotaxin-2 on tumor cells

**DOI:** 10.1186/s13000-020-01061-4

**Published:** 2021-01-12

**Authors:** Rintaro Ohe, Takanobu Kabasawa, Aya Utsunomiya, Yuka Urano, Takumi Kitaoka, Kazushi Suzuki, Naing Ye Aung, Ichiro Kawamura, Katsushi Tajima, Tomoharu Ishiyama, Mitsunori Yamakawa

**Affiliations:** 1 Department of Pathological Diagnostics, Yamagata University Faculty of Medicine, Yamagata University, 2-2-2 Iida-Nishi, Yamagata, 990-9585 Japan; 2 Department of Hematology, Yamagata Prefectural Central Hospital, Yamagata, Japan; 3 Division of Surgery, Yamagata Prefectural Shinjo Hospital, Shinjo, Japan

**Keywords:** Histiocytic sarcoma, Eosinophil, Eotaxin 2, LIGHT, PD-L1

## Abstract

**Background:**

Histiocytic sarcoma (HS) is a rare neoplasm showing morphological and immunophenotypic features of mature tissue histiocytes. We report a patient with nodal HS exhibiting prominent reactive eosinophilic infiltration.

**Case presentation:**

A 68-year-old man presented with intermittent left lower abdominal pain and weight loss over 3 months. A computed tomography scan revealed multiple abdominal nodules. Open biopsy of the mesenteric tumors was performed for definitive diagnosis. Histologically, the tumor was comprised of a diffuse noncohesive proliferation of pleomorphic large cells, including multinucleated cells. Neoplastic cells were positive for histiocytic markers (CD68, CD163, and LIGHT) and PD-L1 but lacked markers of Langerhans cells, follicular dendritic cells, and epithelial cells. Frequent reactive inflammatory cells were intermingled in the background. Interestingly, prominent eosinophilic infiltration was also noted. Spindle neoplastic cells were prone to be present around areas with little to no eosinophilic infiltration and exhibiting fibrosis and lymphatic vessel proliferation. Conversely, polygonal neoplastic cells were prone to be present around areas with relatively large amounts of eosinophilic infiltration without fibrosis or lymphatic vessel proliferation. Immunohistochemically, the tumor cells and reactive eosinophils expressed eotaxin-2 and eotaxin-3, respectively.

**Conclusion:**

We revealed that eotaxins induced the selective migration of eosinophils into tissues in this case. These eosinophils may affect the tumor remodeling and tumor biology characteristics of HS, such as fibrosis and lymphatic vessel proliferation.

**Supplementary Information:**

The online version contains supplementary material available at 10.1186/s13000-020-01061-4.

## Background

Histiocytic sarcoma (HS) is a rare hematopoietic neoplasm derived from non-Langerhans histiocytes that accounts for less than 1% of all hematolymphoid neoplasms [1] and shows morphological and immunophenotypic features of mature tissue histiocytes. This tumor occurs in the skin and connective tissue (35.8%), followed by the lymph nodes (17%), respiratory system (8.2%), and nervous system (6.9%) [2–4]. The neoplastic cells are usually large and round-to-oval in shape [2] and are immunophenotypically positive for histiocytic markers, such as CD4, CD68, CD163, and lysozyme. Somewhat frequently, cases of HS arise from other hematopoietic malignancies, such as follicular lymphoma, chronic lymphocytic leukemia, diffuse large B-cell lymphoma, and B- or T-lymphoblastic leukemia, which have been shown to include secondary HS [4–6]. The prognosis of patients who have concomitant hematological neoplasms (non-Hodgkin lymphoma and acute myeloid leukemia) is relatively poor, and HS has an increased risk of subsequent development in patients with non-Hodgkin lymphoma [4]. Indeed, clonal IgH or TCR rearrangements are found in these secondary HS cases [7]. Recently, the variation rate of the *BRAF* V600E mutation was reported to be low (12%), and HS cases in which complete remission is achieved via molecular targeted therapy are rare [5, 8].

HS tumors are usually accompanied by a variable number of reactive cells, including small lymphocytes, plasma cells, neutrophils, and scant eosinophils [2, 9, 10]. Infiltrating eosinophils are usually not predominant, so no previous reports on HS have paid close attention to the significance of this type of inflammatory cell. Here, we first report a patient with nodal HS exhibiting marked reactive eosinophilic infiltration. This is the first case showing eotaxin-2 (CCL24) expression on HS neoplastic cells themselves, which may induce eosinophil migration in the tumor tissue.

## Case presentation

### Clinical presentation

**Fig. 1:**
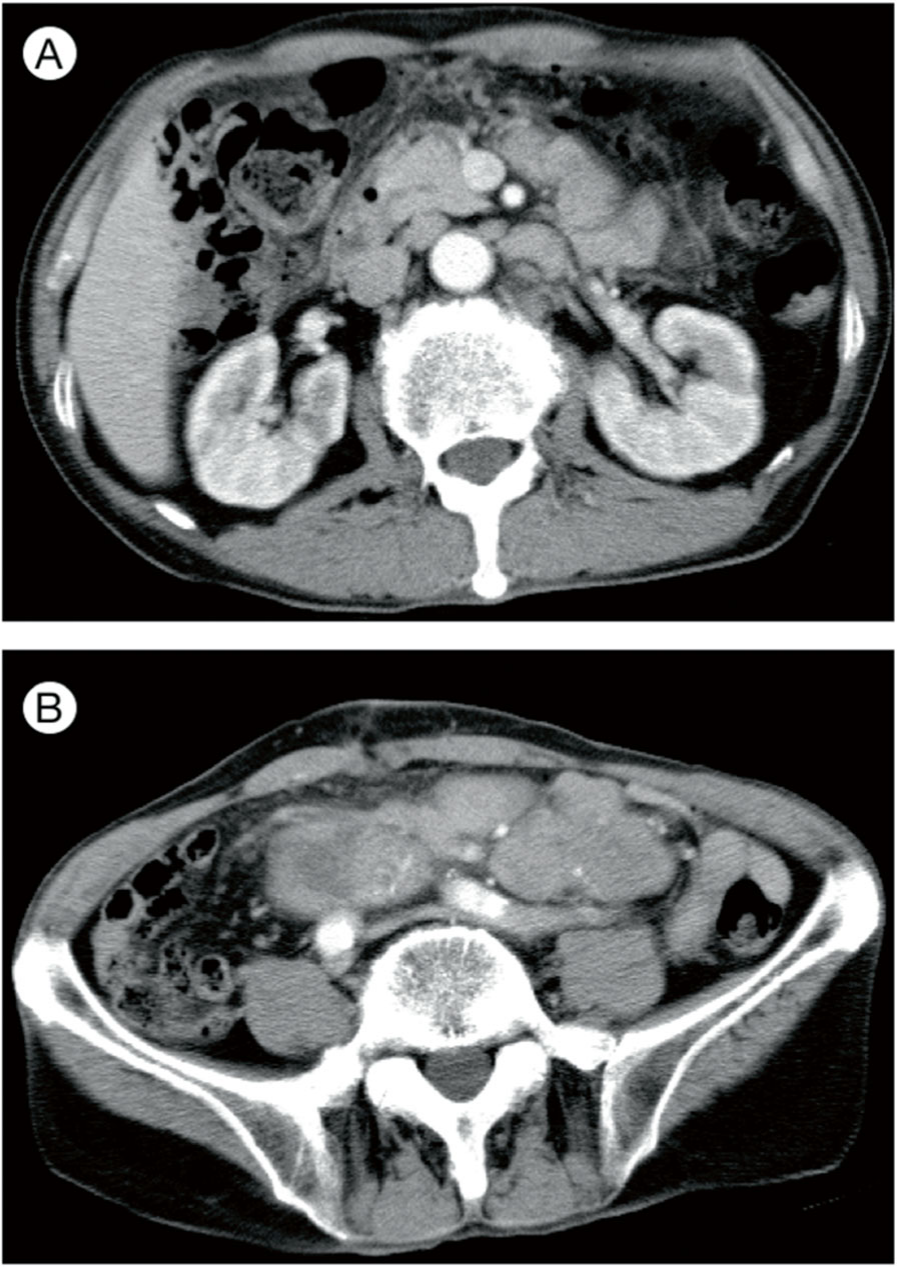
Radiological findings of the abdomen: Abdominal computed tomography scan revealed multiple lymph nodes were swollen in retroperitoneum (**a**) and mesentery (**b**)

A 68-year-old man experienced intermittent left abdominal pain for 3 months. On physical examination, he was afebrile, and superficial lymph node swelling was not detected. The serum level of soluble interleukin-2 receptor was elevated (6020 U/ml). There was no abnormality in the complete blood count or other biochemical examinations. An abdominal computed tomography (CT) scan and Ga-scintigraphy revealed multiple nodules in the retroperitoneum and mesentery (Fig. 1a-b). Hepatosplenomegaly and ascites were absent. An open biopsy revealed multiple nodules, up to 4 cm in the maximal length, involving the mesenteric lymph nodes. He did not elect to be treated with chemotherapy and was given best supportive care. After 25 months of follow-up, he died.

### Cytological and histological findings

**Fig. 2:**
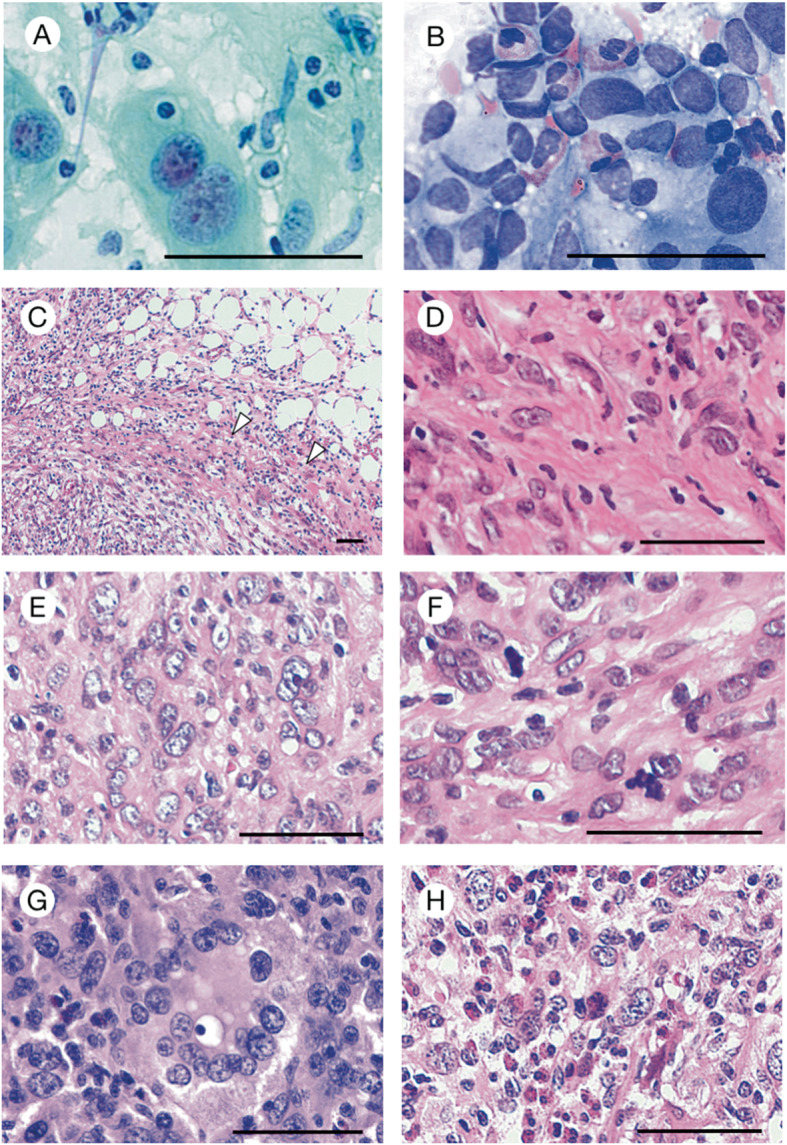
Cytological and histological findings of the lymph node: Mononuclear and multinuclear neoplastic cells were intermingled in an inflammatory background including several eosinophils (**a***, Papanicolaou stain;* **b***, May-Grunwald Giemsa stain*). These were large and round to oval shape, and the nuclei had some nucleoli. An emperipolesis of small lymphocytes was noted. The tumor had a capsule (*Arrowheads*), but neoplastic cells broke through it (**c**). The tumor was composed of large cells which had round to oval or spindle shape with diffuse architecture (**d, e**), and mitoses (**f**) and emperipolesis (**g**) were scattered. Numerous numbers of eosinophils infiltrated into the tumor (**h**). Bars, 50 μm

**Fig. 3:**
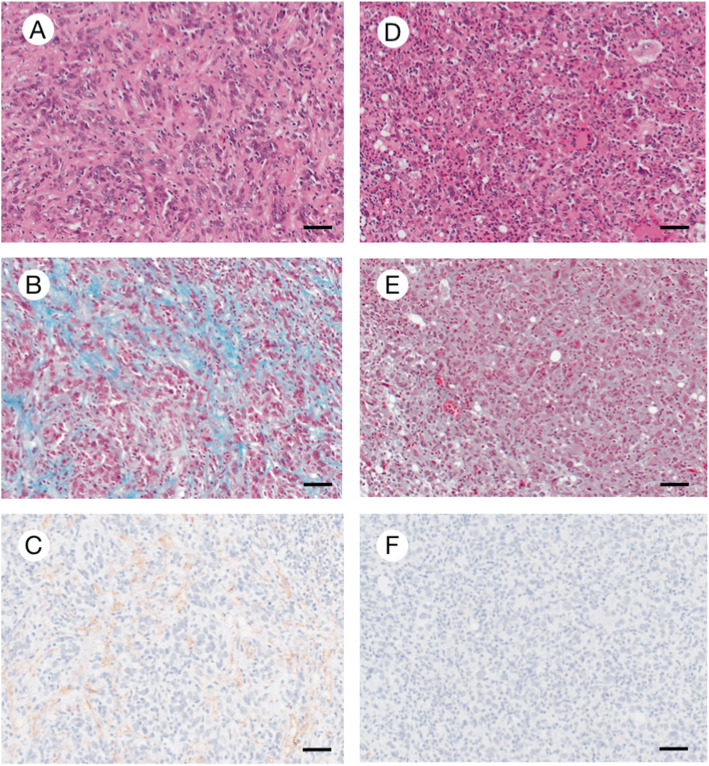
Difference of Histological findings between the areas with and without eosinophilic infiltration: **a-c**; Spindle neoplastic cells (**a**) were prone to present around no or scant eosinophilic infiltration area with fibrosis (**b***, Elastica Masson-Goldner stain)* and D2–40-positive lymphatic vessel proliferation (**c**). **d-f**; Conversely, polygonal neoplastic cells (**d**) were prone to present around relative much eosinophilic infiltration area without evident fibrosis (**e***, Elastica Masson-Goldner stain)* and D2–40-positive lymphatic vessel proliferation (**f**). Bars, 50 μm

Imprints of biopsied tissue specimens stained with Papanicolaou and May-Giemsa were used for cytological examination. Then, the biopsied tissue specimens were fixed in 10% buffered formalin, and paraffin-embedded tissue sections were used for histological examination. On cytological findings, pleomorphic and epithelioid large mononuclear and multinuclear neoplastic cells were intermingled in an inflammatory background including frequent eosinophils (Fig. 2a-b). Nuclei were large and round to oval in shape, and they had some nucleoli. Often, emperipolesis of small lymphocytes was noted. On histological findings, neoplastic cells diffusely proliferated in the whole lymph node and infiltrated over the lymph node capsule (Fig. 2c). The normal architecture of the lymph node was completely affected. The tumor was composed of large (usually > 20 μm) neoplastic cells that were round to oval or spindle in shape and mononuclear or multinucleated with abundant eosinophilic cytoplasm (Fig. 2d-e). There were 1.1 mitoses/high-power field (HPF) on average (Fig. 2f) and emperipolesis (Fig. 2g) with small lymphocytes penetrating neoplastic cells in the same pattern noted in some previous reports [11, 12]. Reactive cells, such as small lymphocytes, plasma cells, neutrophils, benign histiocytes, and numerous eosinophils, infiltrated the neoplastic tissue. The maximum number of eosinophils infiltrating the tumor was 108 cells/HPF (hot spot) (Fig. 2h). Spindle neoplastic cells were prone to present around no or scant eosinophilic infiltration areas showing fibrosis and lymphatic vessel proliferation. On the other hand, polygonal neoplastic cells were prone to present around relatively large eosinophilic infiltration areas without fibrosis or lymphatic vessel proliferation (Fig. 3a-f).

**Fig. 4:**
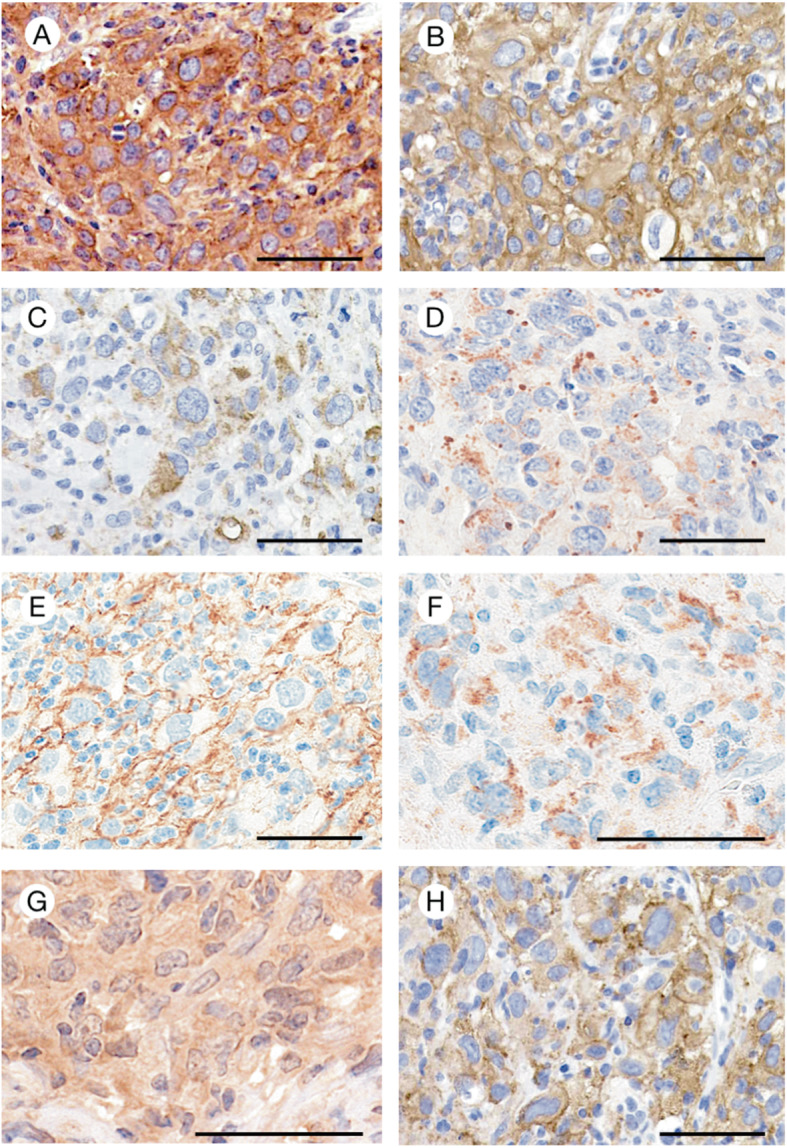
Immunophenotypic finding of the lymph node: Large neoplastic cells were positive for histiocytic markers, such as CD68 (**a**), CD163 (**b**), LN5 (**c**), LIGHT (**d**; clone 4E3, Santa Cruz Biotechnology), and PD-L1 (**e**; clone E1L3N, Cell Signaling Technology). Neoplastic cells were also positive for BRAF V600E (**f**; clone RM8, Abnova), Eotaxin-2 (**g**; clone G-17, Santa Cruz Biotechnology), and DC-SIGN (**h**). Bars, 50 μm

On immunohistochemical findings, neoplastic cells were positive for histiocytic markers such as CD68; CD163; LN5; and homologous to lymphotoxin, inducible expression, competes with herpes simplex virus (HSV) glycoprotein D for binding to HSV entry mediator, a receptor expressed on T lymphocytes (LIGHT, also known as CD258/TNFSF14 [M2b macrophage]), as well as PD-L1 (CD274), BRAF V600E, and eotaxin-2 (Table 1 and Fig. 4a-g). Some tumor cells were positive for lysozyme, LN2, LN3, and DC-SIGN (Fig. 4h). Conversely, neoplastic cells were also negative for eotaxin-3 (CCL26) and other macrophage (heme oxygenase 1 [Mox macrophage], CD205 [DEC205], and CD208 [DC-LAMP]), Langerhans cell, follicular dendritic cell, epithelial cell, lymphocyte, melanoma, neuroendocrine, and mesenchymal cell markers. The Ki-67 labeling index was 31.6%. Numerous eosinophils were positive for eotaxin-3. The results of polymerase chain reaction (PCR) analysis of paraffin-embedded tissues showed a monoclonal band of TCRγ gene rearrangement, but a clonal band of IgH gene rearrangement was not detected. The *BRAF V600E* mutation was not detected by the i-densy IS5320 system (Arkray Inc., Kyoto, Japan) (Supplemental figure).

**Table 1:** Immunohistochemical findings of this case

Markers		Neoplastic cells	Intratumoral eosinophils
Histiocytic marker	CD68	+	–
	CD163	+	–
	LN5	+	–
	LIGHT	+	–
	PD-L1	+	–
	Lysozyme	partially +	–
	LN2	partially +	–
	LN3	partially +	–
	DC-SIGN	partially +	–
	HO-1	–	–
	CD205 (DEC205)	–	–
	CD208 (DC-LAMP)	–	–
Langerhans cell marker	S-100 protein	–	–
	CD1a	–	–
	Langerin	–	–
FDC marker	CD21	–	–
	CD23	–	–
	CD35	–	–
	Clusterin	–	–
Epithelial cell marker	Cytokeratins	–	–
	EMA	–	–
Lymphocyte marker	CD3	–	–
	CD4	–	–
	CD5	–	–
	CD8	–	–
	CD10	–	–
	CD20	–	–
	CD30	–	–
	CD56	–	–
	PAX5	–	–
	PD-1	–	–
Melanoma marker	HMB45	–	–
	Melan A	–	–
Neuroendocrine marker	chromogranin A	–	–
	synaptophysin	–	–
Mesenchymal cell marker	caldesmon	–	–
	desmin	–	–
	α-SMA	–	–
Others	Eotaxin-2	+	+
	Eotaxin-3	–	+

FDC, follicular dendritic cell; LIGHT, homologous to lymphotoxin, inducible expression, competes with herpes simplex virus [HSV] glycoprotein D for binding to HSV entry mediator, a receptor expressed on T lymphocytes]/CD258/TNFSF14; HO-1, heme oxygenase 1

## Discussion and conclusion

HS is a rare neoplasm of mature histiocytes that often has an aggressive clinical course and can arise de novo or from a low-grade B-cell lymphoma [1, 9]. HS usually shows a diffuse architecture involving either nodal or extranodal tissue. Neoplastic cells are large and round to oval with abundant eosinophilic cytoplasm. Neoplastic giant cells or spindle cells can be seen. Hemophagocytosis or emperipolesis by neoplastic cells can be present. There is usually a prominent inflammatory background consisting of neutrophils and lymphocytes. Tumor cells are immunohistochemically positive for CD4, CD11c, CD14, CD45, CD45RO, CD68, CD163, and lysozyme and often or rarely positive for CD15, the S100 protein, and CD56 but negative for markers associated with the B cell lineage, the T cell lineage, follicular dendritic cells (CD21, CD23, and CD35) and Langerhans cells (langerin, and CD1a) as well as CD30, CD34, HMB45, myeloperoxidase, EMA, and cytokeratins [1, 9]. The Ki-67 index varies from 10 to 90% of tumor cells. Our case was morphologically and immunohistochemically compatible with HS.

In HS, a variable number of reactive cells, including small lymphocytes, plasma cells, benign histiocytes, neutrophils, and frequently fewer/scant eosinophils, may be seen [2, 9]. In this case, especially on cytological and histological findings, the maximum number of eosinophils infiltrating the tumor was 108 cells/HPF (hot spot) (Fig. 2f). Therefore, we investigated the mechanism of eosinophilic infiltration of this tumor by utilizing anti-eotaxin-2 and anti-eotaxin-3 antibodies. Eotaxins comprise three types: eotaxin-1 (CCL11), eotaxin-2, and eotaxin-3. Eotaxin-2 and eotaxin-3 have been identified in the human genome and are known to have eosinophil-selective chemoattractant activity [13]. In humans, eotaxin-2 is secreted by various types of cells, including most infiltrating eosinophils as well as other inflammatory cells, epithelial cells, endothelial cells in nasal polyps, and cytokine-stimulated human lung alveolar epithelial cells [14, 15]. Eotaxin-3, and not the other eotaxins, seems to play a major and specific role in sustained severe eosinophil infiltration in asthmatic tissues [16]. Eotaxins are unusual (but not unique) in signaling via a single receptor: C-C motif chemokine receptor 3 (CCR3). CCR3 is found on eosinophils, basophils, mast cells and a subpopulation of Th2 lymphocytes [17]. The eosinophilic infiltration in neoplastic tissue in our case was induced by some chemotactic factors, including eotaxin-2 and eotaxin-3, which are secreted by neoplastic cells and eosinophils, respectively. Namely, this phenomenon may include the secretion of eotaxin-2 from neoplastic cells combined with CCR3 expression on eosinophils. Furthermore, activated eosinophils secrete eotaxin-3. Subsequently, eotaxin-3 may combine with CCR3 expressed on eosinophils and induce the selective migration of numerous eosinophils into tumor tissues.

The neoplastic cells of HS have not been reported to express eotaxin-2. However, there are some lymphoma cases with eotaxin expression. Although a high level of eotaxin mRNA expression was detected in Hodgkin lymphoma, especially nodular sclerosis [18], eotaxin was expressed on fibroblasts rather than Hodgkin/Reed-Sternberg cells themselves [19]. Eotaxin-3 mRNA expression was detected in some cases of follicular lymphoma [20], and multiple reports on cancers associated with eotaxin-2 expression exist [21–23]. In clear cell renal cell carcinoma, patients with high eotaxin-2 expression had poor survival rates [21]. In hepatocellular carcinoma, eotaxin-2 contributes to malignancy via the angiogenesis pathway and indicates a poor prognosis [22]. In colorectal cancer, eotaxin-2 was upregulated in cases with liver metastases [23]. Therefore, eotaxin-2 expression in neoplastic cells may be a poor prognostic factor related to angiogenesis and metastasis.

Via the secretion of various cytokines, tissue eosinophils are involved in diverse biological events, such as host protection against helminths; steady-state development in the mammary glands and intestines; metabolic homeostasis of adipose tissue in coordination with M2 macrophages; tissue remodeling (tissue regeneration and repair, angiogenesis, and fibrosis); cell-mediated immunity (T cell activation and polarization from Th0 to Th1, Th2, and Th17 cells and recruitment of dendritic cells and T cells); humoral immunity (maintenance of bone marrow plasma cells and secretory IgA in the intestines); and cell-cell interactions among dendritic cells, mast cells, and neurons [24]. In addition, eosinophils have ambivalent roles that are both anti- and protumorigenic [25]. In regards to their antitumorigenic role, eosinophils can induce tumor cell death by secreting cytotoxic proteins (major basic protein, eosinophil cationic protein, eosinophil-derived neurotoxin, and granzymes), suppress tumor metastasis by IL-10 and IL-12, and induce cytotoxicity towards tumor cells [25]. Furthermore, eosinophils indirectly promote antitumor immunity by releasing IFNγ and support antitumor immunity by controlling angiogenesis [25]. In regards to their protumorigenic activity, eosinophils inhibit effector T-cell responses and induce suppressive immunity via indoleamine 2,3-dioxygenase [25]. Furthermore, eosinophils may induce tumor cell growth and epithelial mesenchymal transition via epidermal growth factor and transforming growth factor-β1, respectively, and may facilitate metastases by promoting matrix remodeling via the secretion of matrix metalloproteinases 2 and 9 [25]. In our case, no fibrosis or lymphatic vessel proliferation was observed in the area with high eosinophilic infiltration, suggesting that areas with high eosinophil numbers existed in the stage prior to the start of remodeling. Conversely, fibrosis and lymphatic vessel proliferation were observed around areas with no or scant eosinophilic infiltration, suggesting that eosinophil infiltration had already subsided in the areas with extensive fibrosis and lymphatic proliferation. Namely, the increased eosinophilic infiltration in this case would be affected in both the protumorigenic and antitumorigenic activities phases in the tumor microenvironment.

LIGHT can be used to identify murine M2b macrophages but not human M2b macrophages [26]. In this case, neoplastic cells were identified as positive for LIGHT for the first time. This suggests that LIGHT may be useful as an HS marker in humans. The neoplastic cells were also positive for PD-L1 in this case. Although 8 of 10 HS cases showed neoplastic cell expression of PD-L1, these cases were not associated with gains of 9p24.1 [6], unlike classic Hodgkin lymphoma [27]. Furthermore, PD-L1 expression in HS may be associated with the macrophage phenotype and not associated with amplification of 9p24.1 because the staining intensity of PD-L1^+^ neoplastic cells was similar to that of normal macrophages [6]. Therefore, the effect of PD-L1/PD-1 blockade on HS is unknown [6].

In conclusion, we revealed that eotaxins induced the selective migration of eosinophils into tissues in this case. Although the reason why predominant eosinophilic infiltration occurred in this HS case is not clear, these eosinophils may be associated with tissue remodeling processes, such as fibrosis and lymphatic vessel proliferation.

## Supplementary Information

**Additional file 1: Supplemental Figure.** Analysis of *BRAF V600E* mutation of from formalin-fixed paraffin embedded tissue: Since there was only one peak in *BRAF*, the DNA of this case was considered to be wild-type by using the i-densy IS5320 system (Arkray Inc., Kyoto, Japan).

## Data Availability

Is available upon request from the corresponding author.

## Abbreviations

HS: Histiocytic sarcoma
HPF: High-power field
LIGHT: Homologous to lymphotoxin, inducible expression, competes with herpes simplex virus (HSV) glycoprotein D for binding to HSV entry mediator, a receptor expressed on T lymphocytes
PCR: Polymerase chain reaction
CCR3: C-C motif chemokine receptor 3

## References

[1] Jaffe R, Chang KL, Weiss LM, Jaffe ES, Arber DA, Campo E, Harris NL, Quintanilla-Martinez L (2017). Histiocytic sarcoma. Hematopathology.

[2] Weiss LM, Pileri SA, JKC C, CDM F, Swerdlow SH, Campo E, Harris NL (2017). Histiocytic sarcoma. WHO classification of tumours of haematopoietic and lymphoid tissues.

[3] Takahashi E, Nakamura S (2013). Histiocytic sarcoma : an updated literature review based on the 2008 WHO classification. J Clin Exp Hematop.

[4] Kommalapati A, Tella SH, Durkin M, Go RS, Goyal G (2018). Histiocytic sarcoma: a population-based analysis of incidence, demographic disparities, and long-term outcomes. Blood.

[5] Egan C, Nicolae A, Lack J (2020). Genomic profiling of primary histiocytic sarcoma reveals two molecular subgroups. Haematologica.

[6] Shanmugam V, Griffin GK, Jacobsen ED, Fletcher CDM, Sholl LM, Hornick JL (2019). Identification of diverse activating mutations of the RAS-MAPK pathway in histiocytic sarcoma. Mod Pathol.

[7] Vos JA, Abbondanzo SL, Barekman CL, Andriko JW, Miettinen M, Aguilera NS (2005). Histiocytic sarcoma: a study of five cases including the histiocyte marker CD163. Mod Pathol.

[8] Idbaih A, Mokhtari K, Emile JF (2014). Dramatic response of a BRAF V600E-mutated primary CNS histiocytic sarcoma to vemurafenib. Neurology.

[9] Skala SL, Lucas DR, Dewar R (2018). Histiocytic sarcoma: review, discussion of transformation from B-cell lymphoma, and differential diagnosis. Arch Pathol Lab Med.

[10] Stacher E, Beham-Schmid C, Terpe HJ, Simiantonaki N, Popper HH (2009). Pulmonary histiocytic sarcoma mimicking pulmonary Langerhans cell histiocytosis in a young adult presenting with spontaneous pneumothorax: a potential diagnostic pitfall. Virchows Arch.

[11] Nanri A, Katayama E, Imamura T (2020). Cutaneous Histiocytic sarcoma with cellular cannibalism. Am J Dermatopathol.

[12] Shao H, Xi L, Raffeld M (2011). Clonally related histiocytic/dendritic cell sarcoma and chronic lymphocytic leukemia/small lymphocytic lymphoma: a study of seven cases. Mod Pathol.

[13] Zimmermann N, Hershey GK, Foster PS, Rothenberg ME (2003). Chemokines in asthma: cooperative interaction between chemokines and IL-13. J Allergy Clin Immunol.

[14] Heiman AS, Abonyo BO, Darling-Reed SF, Alexander MS (2005). Cytokine-stimulated human lung alveolar epithelial cells release eotaxin-2 (CCL24) and eotaxin-3 (CCL26). J Interf Cytokine Res.

[15] Yao T, Kojima Y, Koyanagi A (2009). Eotaxin-1, −2, and −3 immunoreactivity and protein concentration in the nasal polyps of eosinophilic chronic rhinosinusitis patients. Laryngoscope.

[16] Provost V, Larose MC, Langlois A, Rola-Pleszczynski M, Flamand N, Laviolette M (2013). CCL26/eotaxin-3 is more effective to induce the migration of eosinophils of asthmatics than CCL11/eotaxin-1 and CCL24/eotaxin-2. J Leukoc Biol.

[17] Conroy DM, Williams TJ (2001). Eotaxin and the attraction of eosinophils to the asthmatic lung. Respir Res.

[18] Teruya-Feldstein J, Jaffe ES, Burd PR, Kingma DW, Setsuda JE, Tosato G (1999). Differential chemokine expression in tissues involved by Hodgkin's disease: direct correlation of eotaxin expression and tissue eosinophilia. Blood.

[19] Jundt F, Anagnostopoulos I, Bommert K (1999). Hodgkin/Reed-Sternberg cells induce fibroblasts to secrete eotaxin, a potent chemoattractant for T cells and eosinophils. Blood.

[20] Shiraishi J, Nakagawa Y, Kurata M (2008). Follicular lymphoma with marked infiltration of eosinophils. Pathol Int.

[21] Wang C, Wang Y, Hong T (2020). Blocking the autocrine regulatory loop of Gankyrin/STAT3/CCL24/CCR3 impairs the progression and pazopanib resistance of clear cell renal cell carcinoma. Cell Death Dis.

[22] Jin L, Liu WR, Tian MX (2017). CCL24 contributes to HCC malignancy via RhoB- VEGFA-VEGFR2 angiogenesis pathway and indicates poor prognosis. Oncotarget.

[23] Cheadle EJ, Riyad K, Subar D (2007). Eotaxin-2 and colorectal cancer: a potential target for immune therapy. Clin Cancer Res.

[24] Weller PF, Spencer LA (2017). Functions of tissue-resident eosinophils. Nat Rev Immunol.

[25] Grisaru-Tal S, Itan M, Klion AD, Munitz A (2020). A new dawn for eosinophils in the tumour microenvironment. Nat Rev Cancer.

[26] Wang LX, Zhang SX, Wu HJ, Rong XL, Guo J (2019). M2b macrophage polarization and its roles in diseases. J Leukoc Biol.

[27] Xie W, Medeiros LJ, Li S, Yin CC, Khoury JD, Xu J. PD-1/PD-L1 pathway and its blockade in patients with classic Hodgkin lymphoma and non-Hodgkin large-cell lymphomas. Curr Hematol Malig Rep. 2020. 10.1007/s11899-020-00589-y 32394185

